# Association between Ambient Air Pollution and Emergency Room Visits for Pediatric Respiratory Diseases: The Impact of COVID-19 Pandemic

**DOI:** 10.3390/toxics10050247

**Published:** 2022-05-14

**Authors:** Chi-Yung Cheng, Yu-Lun Tseng, Kuo-Chen Huang, I-Min Chiu, Hsiu-Yung Pan, Fu-Jen Cheng

**Affiliations:** 1 Department of Emergency Medicine, Kaohsiung Chang Gung Memorial Hospital, College of Medicine, Chang Gung University, Kaohsiung 833, Taiwan; qzsecawsxd@cgmh.org.tw (C.-Y.C.); bluescratch7@gmail.com (K.-C.H.); outofray@hotmail.com (I.-M.C.); gettingfat720@gmail.com (H.-Y.P.); 2 College of Medicine, Chang Gung University, Taoyuan 333, Taiwan; 3 Department of Computer Science and Engineering, National Sun Yat-sen University, 70, Lian-Hai Road, Kaohsiung 804, Taiwan; 4 Institute of Environmental Engineering, National Sun Yat-sen University, 70, Lian-Hai Road, Kaohsiung 804, Taiwan; yulun1129@g-mail.nsysu.edu.tw

**Keywords:** particulate matter, air pollution, respiratory diseases, emergency department, pediatric, COVID-19 pandemic

## Abstract

The level and composition of air pollution have changed during the coronavirus disease 2019 (COVID-19) pandemic. However, the association between air pollution and pediatric respiratory disease emergency department (ED) visits during the COVID-19 pandemic remains unclear. The study was retrospectively conducted between 2017 and 2020 in Kaohsiung, Taiwan, from 1 January 2020 to 1 May 2020, defined as the period of the COVID-19 pandemic, and 1 January 2017 to 31 May 2019, defined as the pre-COVID-19 pandemic period. We enrolled patients under 17 years old who visited the ED in a medical center and were diagnosed with respiratory diseases such as pneumonia, asthma, bronchitis, and acute pharyngitis. Measurements of particulate matter (PM) with aerodynamic diameters of <10 μm (PM_10_) and < 2.5 μm (PM_2.5_), nitrogen dioxide (NO_2_), and Ozone (O_3_) were collected. During the COVID-19 pandemic, an increase in the interquartile range of PM_2.5_, PM_10_, and NO_2_ levels was associated with increases of 72.5% (95% confidence interval [CI], 50.5–97.7%), 98.0% (95% CI, 70.7–129.6%), and 54.7% (95% CI, 38.7–72.6%), respectively, in the risk of pediatric respiratory disease ED visits on lag 1, which were greater than those in the pre-COVID-19 pandemic period. After adjusting for temperature and humidity, the risk of pediatric respiratory diseases after exposure to PM_2.5_ (inter *p* = 0.001) and PM_10_ (inter *p* < 0.001) was higher during the COVID-19 pandemic. PM_2.5_, PM_10_, and NO_2_ may play important roles in pediatric respiratory events in Kaohsiung, Taiwan. Compared with the pre-COVID-19 pandemic period, the levels of PM_2.5_ and PM_10_ were lower; however, the levels were related to a greater increase in ED during the COVID-19 pandemic.

## 1. Introduction

In early 2020, coronavirus disease 2019 (COVID-19) spread rapidly worldwide. From 21 January to 30 April, there were 429 confirmed COVID-19 cases and six deaths in Taiwan (https://www.cdc.gov.tw/en/Disease/SubIndex/, accessed on 27 June 2021). The COVID-19 pandemic created an unparalleled burden on the healthcare system, especially frontline emergency department (ED) services. During the outbreak, the number of ED visits decreased significantly, with an approximately 40–60% decrease in the number of visits in the USA [1]. Regarding cardiovascular diseases, compared with 2019, there was a 20% decrease in the number of stroke-related ED visits and a 26% decrease in hospital admissions for acute coronary syndrome (ACS) [2]. In Taiwan, a 25–30% reduction in ED visits was also mentioned [3]. However, the disease causing the largest reduction in ED visits remains unclear. Sung et al. revealed that the patient number of ED visits with triage level 1 remained unvarying, while patients with triage levels 2–5 showed a decreasing trend during the COVID-19 pandemic [4]. In contrast, another study showed increased medical out-of-hospital cardiac arrest (OHCA) during the same period [5]. Among all the pediatric ED visits in an Italian hospital, the proportion of upper respiratory diseases reduced the most [6].

Simultaneously, air pollution conditions also changed during the COVID-19 pandemic. During that period, some countries lock down social and industrial activities globally. According to data from the National Aeronautics and Space Administration (NASA) and European Space Agency (ESA), there was a 20–30% reduction in nitrate dioxide (NO_2_) in the USA, Europe, and China [7]. Regarding particulate matter (PM), Mahato et al. revealed 60% and 39% decreases in PM_10_ (PM with an aerodynamic diameter <10 μm) and PM_2.5_ (PM with an aerodynamic diameter <2.5 μm) in India [8]. In Taiwan, PM_2.5_ levels also decreased during the COVID-19 pandemic because of reduced long-distance transport of air pollutants from China and reduced industrial activity [9]. 

Many epidemiologic studies have reported the hazardous effects of air pollution exposure on human health, such as cardiovascular diseases, respiratory diseases, and out-of-hospital cardiac arrest (OHCA) [10,11,12,13]. The hazardous effects of air pollution appear to exhibit regional and seasonal heterogeneity. These regional and seasonal variations are partly explained by community characteristics, such as weather conditions and the proportion of elderly residents [14,15]. Another possible reason is that different constituents of PM_2.5_ might have different health effects. For example, organic carbon was found to be associated with the risk of ischemic stroke-related ED visits. In contrast, elemental carbon was found to be associated with chronic obstructive pulmonary disease (COPD)-related ED visits [16,17]. Furthermore, PM_2.5_ components also changed during the COVID-19 pandemic [18]. 

Epidemiological evidence implied that environmental exposure such as air pollutants and smoking could have an important impact on the severity and occurrence of COVID-19 infection [19,20]. During the COVID-19 pandemic, many countries have undertaken restrictions on social gatherings, public transportation, and self-protective equipment such as mask-wearing. The association between air pollution and pediatric respiratory disease-related ED visits during the COVID-19 pandemic remains unclear. Although there were decreases in air pollution during the COVID-19 lockdown, air pollution can increase the severity of COVID infection by undermining the individual immune response and aggravating predisposing chronic diseases [21]. McAuley et al. showed the events of COPD acute exacerbation were increased during the COVID pandemic [22]. Fan et al. found a lower risk of severe asthma exacerbations but an increased frequency of mild asthma exacerbations [23]. Taquechel et al. revealed reduced hospital admissions with decreased systemic steroid prescriptions for pediatric asthma in Philadelphia [24]. Moreover, the health effects of the altered composition of PM_2.5_ during the COVID-19 pandemic are not yet understood. The purposes of this study are as follows:

(1) To evaluate the effects of short-term exposure to PM_2.5_ and other air pollutants on pediatric respiratory disease ED visits.
(2) To explore the different hazard effects of PM_2.5_ and other air pollutants on pediatric respiratory diseases before and during the COVID-19 pandemic.

## 2. Methods

### 2.1. Study Population

The COVID-19 pandemic was defined as the period from 1 January 2020 to 31 May 2020. The pre-COVID-19 pandemic period was selected from 1 January 2017 to 31 May 2019 to avoid seasonal effects. This retrospective observational study was conducted between 1 January 2017 and 31 May 2020 in an urban tertiary medical center with an average of 73,000 ED visits per year. We enrolled patients under 17 years of age who visited the ED with a principal diagnosis of “pneumonia” (International Classification of Diseases, tenth revision [ICD-10]: J18),” asthma” (ICD-10: J45), “bronchitis” (ICD-10: J40), and “acute pharyngitis” (ICD-10: J02), and “upper respiratory tract infection (URI)” (ICD-10: J00-06). After two trained emergency physicians (EPs) review, medical records, including demographic factors, such as age, sex, address, and time of ED visits, were obtained from the ED database. The Institutional Review Board of Chang Gung Medical Foundation approved this study (IRB number 202001641B0 approved on 24 April 2020) according to the guidelines of the Declaration of Helsinki. Informed consent was not required for this study owing to its retrospective nature.

### 2.2. Pollutant and Meteorological Data

Air pollutant data and meteorological conditions were acquired from 11 air quality monitoring stations established in Kaohsiung City in 1994 by the Taiwanese Environmental Protection Administration (EPA). The hourly concentrations of four “criteria” pollutants, including PM_10_ (by beta-ray absorption), PM_2.5_ (by beta-ray absorption), NO_2_ (by ultraviolet fluorescence), and O_3_ (by ultraviolet photometry), were obtained during the study period. Weather conditions, including temperature and relative humidity, were also recorded at the monitoring stations. The daily average concentrations of air pollutants and the weather conditions were then calculated. 

### 2.3. Statistical Method

We used a time-stratified case-crossover study design, an alternative design of the Poisson time-series regression model, to analyze the health effects of short-term exposure, as described in our previous studies [10,25]. A case-crossover study design is a special type of case-control study, and within-subject comparisons were performed between case and control periods [26,27]. Time stratification was performed to select referent days as the days falling on the same day of the week (one case day with three to four control days) within the same month as the index day [28]. The day of the pediatric respiratory disease ED visit was defined as lag 0, the day before the episode as lag 1, and the day before lag 1 as lag 2. The levels of air pollution during the case period were compared with those on all referent days. The effect of air pollutants on pediatric respiratory diseases was investigated from lag 0 to lag 3. Conditional logistic regression was used to estimate the odds ratios (ORs) and 95% confidence intervals (CIs) of air pollutants on pediatric respiratory diseases. Subgroup analyses and interaction p values were also calculated to analyze the effect of the COVID-19 pandemic. Temperature and relative humidity were included as confounding factors in the model. Potential nonlinear relationships between air temperature, humidity, and pediatric respiratory diseases were determined using Akaike’s information criterion (AIC) [29]. We used the SAS macro “lgtphcurv9”, which implements a natural cubic spline methodology to fit potential nonlinear response curves in logistic regression models for case-control studies [30]. ORs were calculated based on interquartile range (IQR) increments of PM_10_, PM_2.5_, and other gaseous pollutants. The significance criterion was set at *p* < 0.05. All statistical analyses were performed using SAS software version 9.3.

## 3. Results

During the study period, 11,760 pediatric patients visited the ED with respiratory diseases. A total of 1364 patients were excluded from the analysis because they did not reside in Kaohsiung City; the remaining 10,396 patients were included for further analysis. The demographic characteristics of the 10,396 patients are listed in Table 1. There were 5924 (57.0%) male patients, with a mean age of 4.6 years. In total, 1405 (13.5%) patients visited the ED during the COVID-19 pandemic. Demographic characteristics of pre-COVID-19 and during COVID-19 period are listed in Table S1.

The weather and air pollution conditions during the study period in Kaohsiung are summarized in Table 2. The average PM_2.5_, PM_10_, and NO_2_ concentrations during the COVID-19 pandemic were 20.4 µg/m^3^, 40.8 µg/m^3^, and 13.3 parts per billion (ppb), respectively, significantly lower than the those in the pre-COVID-19 pandemic period. 

Table 3 shows the Spearman correlation coefficients for the air pollutants and weather conditions. PM_2.5_ levels highly correlated with PM_10_ (*r* = 0.939, *p* < 0.001) and NO_2_ levels (*r* = 0.799, *p* < 0.001), and moderately correlated with O_3_ levels (*r* = 0.539, *p* < 0.001).

**Figure 1:**
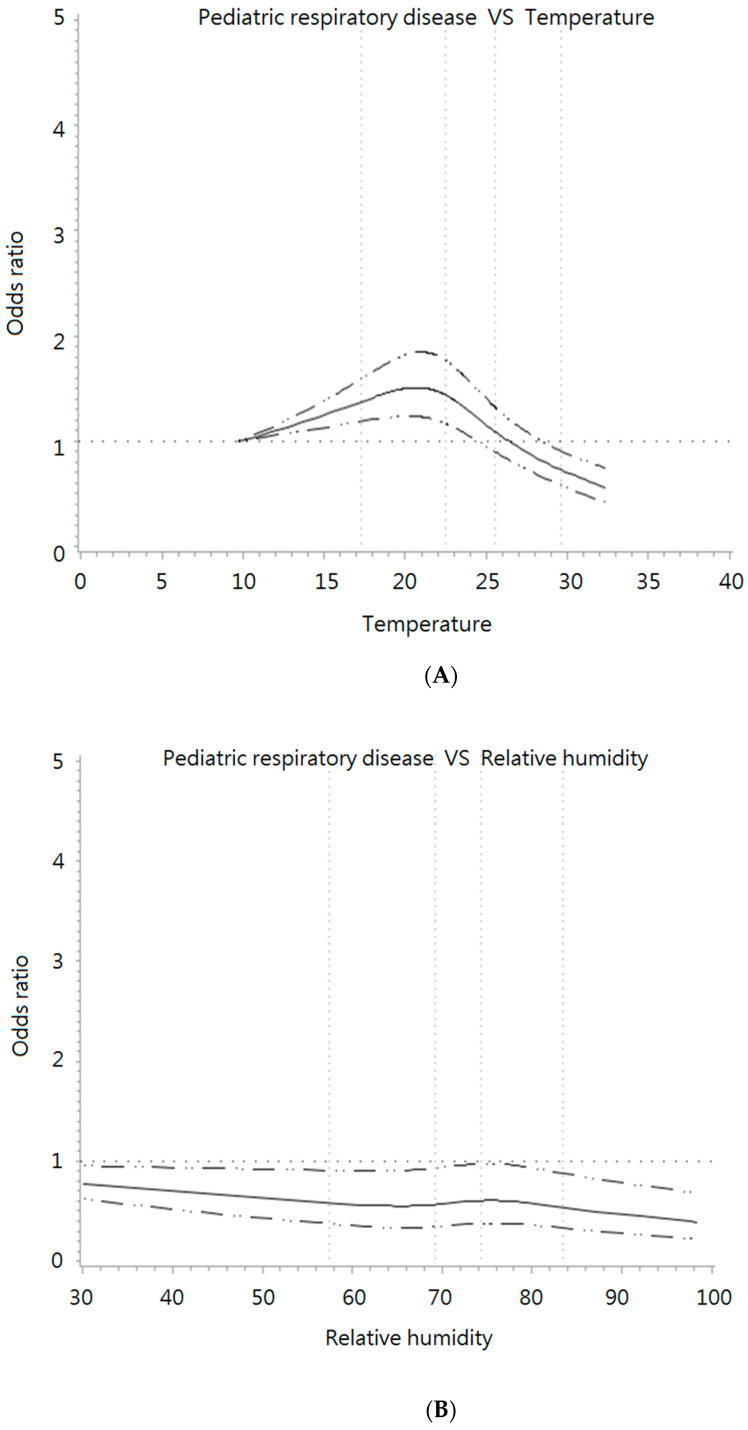
Restricted cubic spline for (**A**) temperature and (**B**) relative humidity.

Before performing conditional logistic regression, potential nonlinear relationships between temperature, humidity, and pediatric respiratory diseases were evaluated using AIC. For temperature, the AIC value of the spline model was 28,629.444, which was better than that of the linear model (AIC = 28,732.096, *p* < 0.001). With respect to relative humidity, the spline model was also better than the linear model (*p* = 0.002), with AIC values of 28,770.224 and 28,783.179, respectively. Therefore, the spline model was used based on the results of the AIC and knots for humidity and temperature [31]. Figure 1 shows the restricted cubic spline and knots for temperature and relative humidity. 

The reference exposure values for temperature and relative humidity were set at 10 °C and 40%, respectively, including four knots. 

**Figure 2:**
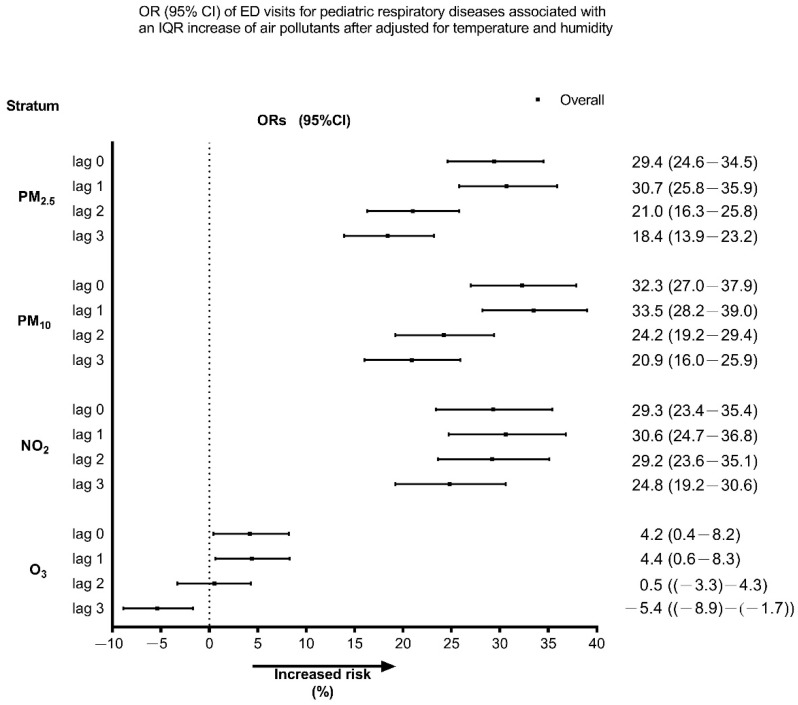
Odds ratios (ORs) and 95% confidence intervals (CIs) for pediatric respiratory disease-related ED visits associated with IQR increments in each air pollutant during the study period, with adjustments for temperature and humidity. ED, emergency department; IQR, interquartile range.

Figure 2 shows the effect of air pollutants on ED visits for pediatric respiratory diseases during the study period. An IQR increment in PM_2.5_, PM_10_, NO_2_, and O_3_ was associated with an increase of 30.7% (95% CI, 25.8–35.9%), 33.5% (95% CI, 28.2–39.0%), 30.6% (95% CI, 24.7–36.8%), and 4.4% (95% CI, 0.6–8.3%) in the risk of pediatric respiratory disease-related ED visits on lag 1, respectively. We observed that the greatest effect occurred on lag 1 and gradually decreased. During the COVID-19 pandemic, an increased IQR for PM_2.5_, PM_10_, and NO_2_ levels was associated with 72.5% (95% CI, 50.5–97.7%), 98.0% (95% CI, 70.7–129.6%), and 54.7% (95% CI, 38.7–72.6%), respectively, increased risk of pediatric respiratory disease-related ED visits on lag 1, which was greater than those during the pre-COVID-19 pandemic period (Supplementary Figure S1).

Two-pollutant model analysis was then performed to determine which individual air pollutants had a greater effect on pediatric respiratory disease-related ED visits, independent of the effects of the other pollutants. The results are presented in Table 4. An IQR increase in PM_2.5_ was significantly related to ED visits for pediatric respiratory diseases after adjustment for PM_10_ (OR = 1.128; 95% CI, 1.055–1.207), NO_2_ (OR = 1.186; 95% CI, 1.138–1.236), and O_3_ (OR = 1.259; 95% CI, 1.212–1.308). The impact of PM_10_ and NO_2_ on pediatric respiratory disease also achieved statistical significance after adjusting for PM_2.5_.

**Figure 3:**
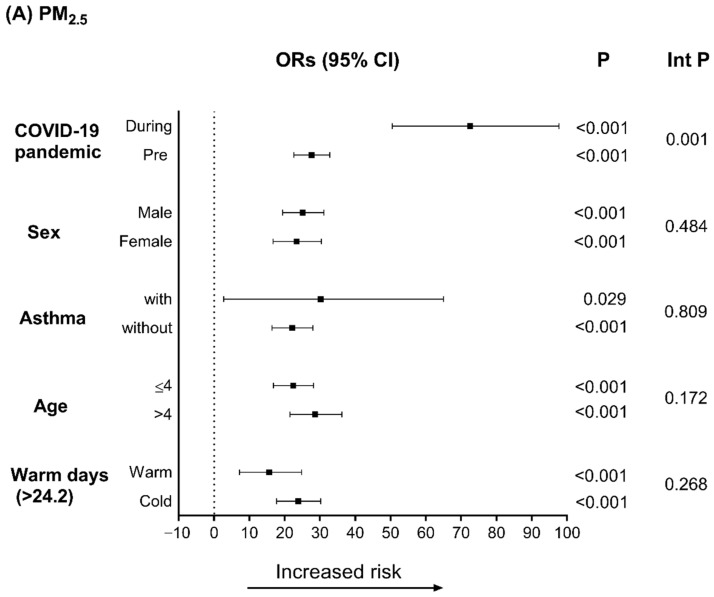

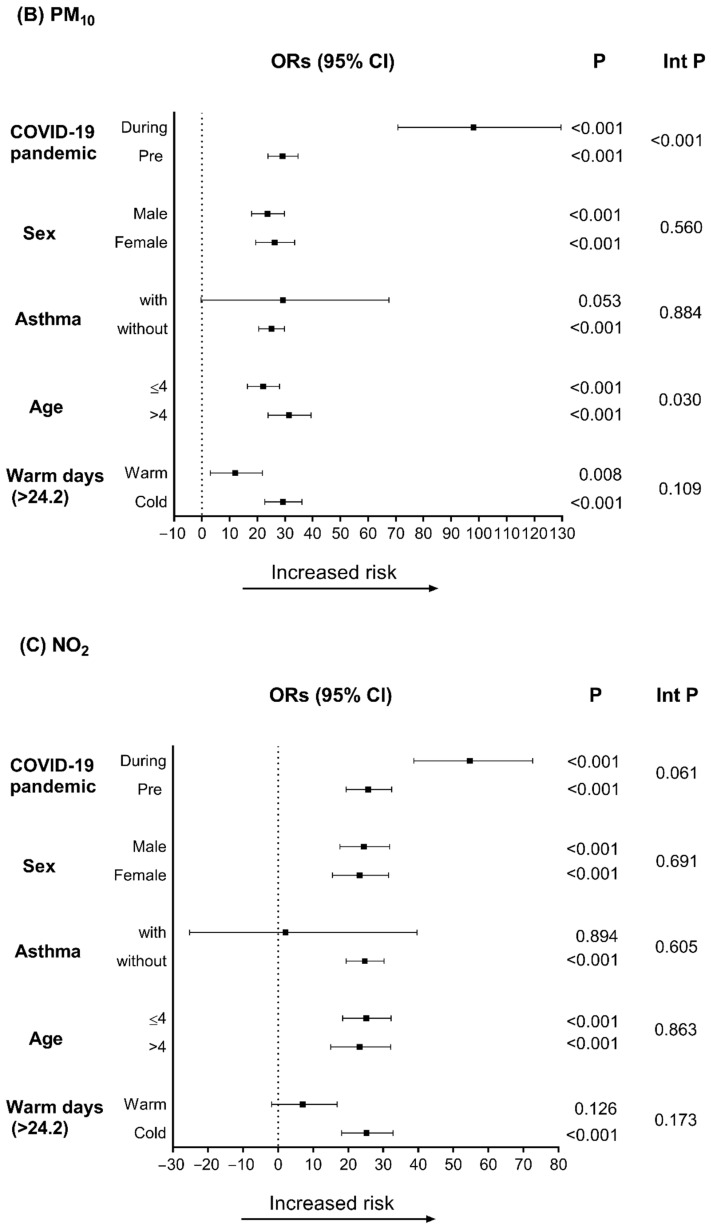
Odds ratios (ORs) for IQR increments in (**A**) PM_2.5_, (**B**) PM_10_, and (**C**) NO_2_ on lag 1 after adjustment for temperature and humidity. Int p, interaction *p*-value.

Figure 3 shows the results of the stratified analysis used to elucidate the effects of PM_2.5_, PM_10_, and NO_2_ on pediatric respiratory diseases according to different periods and demographic factors on lag 1. As shown in Figure 3A,B, after adjusting for temperature and humidity, the risk of pediatric respiratory diseases after exposure to PM_2.5_ (inter *p* = 0.001) and PM_10_ (inter *p* < 0.001) was higher during the COVID-19 pandemic. Figure 3B also shows that older children (>4 years) were more sensitive to the harmful effects of PM_10_ (inter *p* = 0.03). 

## 4. Discussion

In this study, we estimated the effects of air pollutants on pediatric respiratory diseases. We found that PM_2.5_, PM_10_, and NO_2_ may be significantly associated with the risk of pediatric respiratory diseases in Kaohsiung, Taiwan. Furthermore, the hazardous effects of PM_2.5_ and PM_10_ were greater during the COVID-19 pandemic. Older children (>4 years) were more susceptible to PM_10_-related respiratory diseases. 

Epidemiological studies have shown that ambient air pollution is associated with adverse effects on pediatric respiratory diseases, including upper respiratory infections, asthma, bronchitis, and pneumonia [32,33,34]. For URI, Liu et al. found that PM_2.5_ was associated with the risk of URI hospitalization [32]; Xiao et al. demonstrated the hazard effect of O_3_, NO_2_, and PM_2.5_ on URI ED visits [33]. Bono et al. collected data from 21,793 ED admissions for respiratory diseases, including 17,684 patients with URI. They found the impact of NO_2_ on respiratory diseases ED admission, but the effect of PM_2.5_ did not achieve statistical significance [35]. The disparities in these previous studies might be explained by different PM_2.5_ emission sources. Huang et al. collected PM_2.5_ data and analyzed their sources; they concluded that PM_2.5_ from metals and natural gas was positively related to the risk of pneumonia, asthma, and URI ED visits [36]. 

Similarly, previous studies also found a positive association between air pollution and pediatric respiratory diseases. Cheng et al. demonstrated that short-term exposure to PM_2.5_, PM_10_, NO_2_, and SO_2_ three  days before the event increased the odds of pediatric pneumonia by 14.0%, 10.9%, 14.1%, and 4.5%, respectively [10]. Lv et al. found that PM_2.5_ concentrations the day before hospital admission and PM_10_ concentrations 2 days before hospital admission were associated with an increased risk of pediatric hospital admission [37]. Wu et al. assessed air pollution and weather conditions in childhood asthma. They confirmed that exposure to PM_2.5_ and PM_10_ within the past two weeks significantly elevated the risk of exacerbation and decreased the disease control rate [38]. Hwang et al. reported that children were susceptible to the effect of PM_2.5_, especially at lag 0 and lag 1, on asthma-related ED visits [39]. Similarly, we also found that PM_2.5_, PM_10_, and NO_2_ levels were significantly associated with pediatric respiratory diseases, with the greatest effect occurring on lag 1 and gradually decreasing. However, the difference in pediatric respiratory disease at different lag times might be because of variations in ambient air pollution and its components and geographical and seasonal effects. In addition, air pollution policies may play a role in the variation in ambient air pollution risk.

During the COVID-19 pandemic, from February to April 2020, many countries announced first-level responses to major public health emergencies. For the “lockdown period,” many places implemented national efforts limiting travel and social interaction, such as government-imposed stay-at-home orders, reducing human and industrial activity, and closing non-essential businesses and schools. The widespread changes in human behavior and non-pharmaceutical interventions may have significantly reduced emissions from vehicle exhausts and industrial production. The unprecedented actions taken to mitigate the spread of the disease have created large-scale behavioral changes in air quality. Otmani et al. found that the change in the concentrations recorded before and during the COVID-19 pandemic were 75%, 49%, and 96% reductions for PM_10_, SO_2_, and NO_2_, respectively, in Morocco [40]. Yin et al. demonstrated that the COVID-19 lockdown caused a significant reduction in pollutants, including PM_2.5_, PM_10_, NO_2_, SO_2_, and CO concentrations, but not O_3_ [41]. In Spain, the reduction in NO_2_ concentration during the COVID-19 lockdown was approximately 50–62%. The maximum hourly peak values showed a reduction with ratios of approximately 1.2 to 1.7. Traffic from internal combustion motor vehicles is the most important source of polluting emissions in these cities [42]. In the United Kingdom, a significant reduction of mean NO_2_ concentrations of 35.13% at background and 40.82% at traffic sites was observed [43]. In New York City, a city-wide 23% improvement in PM_2.5_ was detected [44]. Xu et al. indicated that the levels of PM_2.5_, PM_10_, NO_2_, SO_2_, and CO were 30.1%, 40.5%, 61.4%, 33.4%, and 27.9% lower, respectively, during February 2020 compared to those in February 2017–2019 in Central China [45]. Industrial production and vehicle exhaust emissions were reduced due to strict epidemic prevention and control actions, leading to significant reductions in NO_2_ and PM_10_ levels. The trend in O_3_ concentration was opposite to that of other air pollutants (PM_2.5_, PM_10_, NO_2_, SO_2_, and CO). One potential reason for increased O_3_ generation may be increased global temperature, leading to intensified photochemical reactions. In addition, a lower concentration of NO_2_ hindered the reaction of NO and O_3_, which led to an increase in O_3_ levels [45]. Similar to previous studies, we also found that the average PM_2.5_, PM_10_, and NO_2_ concentrations during the COVID-19 pandemic were significantly lower than those during the pre-COVID-19 period.

In the present study, although the PM_2.5_ and PM_10_ levels were lower than those in the pre-COVID-19 period, the health effects on pediatric respiratory disease ED visits were greater during the COVID-19 pandemic. Fan et al. also demonstrated that the prevalence of asthma exacerbations did not decrease while the air quality was improved during the COVID-19 pandemic [23]. In contrast, Dias et al. highlighted policies for COVID-19 prevention could significantly reduce in hospitalization mortality due to pediatric pneumonia [46]. This may be because PM is a heterogeneous chemical mixture of solids and liquids containing organic carbon (OC), elemental carbon (EC), inorganic salts, and metals. The toxicity of the constituents and sources of ambient particles are considered to be related to different health outcomes [47,48,49]. Ostro et al. examined the association between specific diseases and different sources of PM_2.5_, including vehicle emissions, soil, biomass burning aerosols, and secondary nitrate and sulfate sources. Vehicular emissions are associated with cardiovascular diseases, such as myocardial infarction and dysrhythmia. In contrast, vehicular emissions, biomass burning, and soil sources were associated with respiratory diseases. The soil source, which mainly consists of resuspended road dust, had the highest risk ratio for asthma [47]. Using two years of daily PM_2.5_ component measurements (including ions, carbon, OC, and EC), Sarnat et al. found robust associations of 17α(H),21β(H)-hopane with cardiovascular disease, EC with congestive heart failure, and ozone with respiratory disease [48]. Peng et al. demonstrated that an elevated IQR for organ carbon increased the risk of emergency hospital admissions in respiratory disease by 1.01, and an elevated IQR level for elemental carbon increased emergency hospital admissions for cardiovascular disease by 0.8 [49]. Therefore, changes in exposure to various sources and compositions of air pollution may be associated with changes in target human health. 

The present study has some limitations. First, individuals were identified in a coastal industrial city with a tropical monsoon climate; the source of ambient air pollutants and the constituent material may be different from those in other areas. Second, personal protective equipment, including air purifiers and face masks, pollutants monitoring, and feedback, could potentially decrease exposure to pollutants [50]. Third, the diagnosis of “pneumonia”, “URI”, “bronchitis” and “asthma” was based on clinical judgment. Diagnosis made by different emergency physicians might impact the data we collected. In addition, we did not analyze the impact of indoor air contaminants and secondhand smoking on respiratory pathologies [51,52]. Finally, the study was conducted in a single tertiary medical center, which limited the sample size and ethnic diversity. Further studies should be conducted in more regions, including more ethnic diversity, to analyze the influence of personal protective equipment.

## 5. Conclusions

PM_2.5_, PM_10_, and NO_2_ may play important roles in pediatric asthma events in Kaohsiung, Taiwan. Compared with the pre-COVID-19 pandemic period, the levels of PM_2.5_ and PM_10_ were lower, but their effects on pediatric respiratory disease-related ED visits were greater during the COVID-19 pandemic. Older children (>4 years old) were more susceptible to PM_10_ and respiratory diseases.

## Figures and Tables

**Table 1:** Demographic characteristics of patients.

All	Number = 10,396	
Demographic characteristics of patients		%
Age (mean ± standard deviation)	4.6 ± 3.7	
Male	5924	57.0
Past medical history of respiratory disease	252	2.4
During COVID-19 pandemic	1405	13.5
Onset on warm days (>24.2 °C)	4809	46.3

**Table 2:** Summary statistics of meteorological factors and air pollutants during the study period in Kaohsiung.

	Percentiles			Mean	Pre-COVID-19 (Mean ± SD)	During COVID-19 (Mean ± SD)	*p*	IQR
	25%	50%	75%					
PM_2.5_ (µg/m^3^)	17.4	27.5	36.1	27.4	30.2 ± 13.0	20.4 ± 11.0	<0.001	18.7
PM_10_ (µg/m3)	39.7	55.3	71.0	55.8	61.8 ± 22.7	40.8 ± 18.5	<0.001	31.3
NO_2_ (ppb)	11.5	15.4	19.7	15.8	16.8 ± 5.3	13.3 ± 5.0	<0.001	8.2
O_3_ (ppb)	22.9	31.3	39.3	31.6	29.8 ± 11.5	31.8 ± 11.0	0.268	16.4
Temperature (°C)	21.3	24.2	27.3	24.1	23.9 ± 3.8	24.7 ± 4.1	0.011	6.0
Humidity (%)	69.3	72.7	75.9	72.4	72.5 ± 6.5	72.3 ± 6.5	0.844	6.6

SD, standard deviation; IQR, interquartile range.

**Table 3:** Spearman correlation coefficients for air pollutants and weather conditions during the study period.

	PM_2.5_	PM_10_	NO_2_	O_3_	Temp	Humidity
PM_2.5_	1.000	0.939	0.799	0.539	−0.659	−0.272
PM_10_		1.000	0.790	0.515	−0.622	−0.350
NO_2_			1.000	0.260	−0.819	−0.177
O_3_				1.000	−0.223	−0.413
Temperature					1.000	0.175
Humidity						1.000

**Table 4:** OR (95% CI) of respiratory diseases ED visits for each interquartile range change in two-pollutant models.

	OR (95% CI) of Respiratory Disease-Related ED Visits for Each Interquartile Range Change in Two-Pollutant Models after Adjusting for Temperature and Humidity			
	Adjust PM_2.5_	Adjust PM_10_	Adjust NO_2_	Adjust O_3_
PM_2.5_		1.128 (1.055–1.207)	1.186 (1.138–1.236)	1.259 (1.212–1.308)
PM_10_	1.125 (1.050–1.204)		1.189 (1.139–1.240)	1.258 (1.211–1.307)
NO_2_	1.119 (1.065–1.176)	1.118 (1.064–1.176)		1.240 (1.188–1.295)
O_3_	0.965 (0.929–1.004)	0.975 (0.939–1.013)	1.050 (1.013–1.089)	

## Data Availability

Restrictions apply to the availability of these data. Data was obtained from Chang Gung Research Database and is available from corresponding author with the permission.

## Supplementary Materials

The following are available online at https://www.mdpi.com/article/10.3390/toxics10050247/s1, Figure S1: Odds ratios (ORs) and 95% confidence intervals (CIs) for pediatric respiratory dis-ease-related ED visits associated with IQR increments in each air pollutant, pre- COVID-19 pandemic (A) and during the COVID-19 pandemic (B), with adjustments for temperature and humidity. ED, emergency depart-ment; IQR, interquartile range, Table S1: Demographic characteristics and diagnoses during study period.
