# Supplementary material for: Association between Ambient Air Pollution and Emergency Room Visits for Pediatric Respiratory Diseases: The Impact of COVID-19 Pandemic
Source: Toxics. 2022 May 14;10(5):247. doi: 10.3390/toxics10050247 (PMC9146083; doi:10.3390/toxics10050247)
Supplement: Supplementary file 1 [file toxics-10-00247-s001.zip › toxics-1677573-SI.pdf]

toxics

MDPI

Supplementary Materials: Association between Ambient Air Pollution and Emergency Room Visits for Pediatric Respiratory Diseases: The Impact of COVID-19 Pandemic

Chi-Yung Cheng, Yu-Lun Tseng, Kuo-Chen Huang, I-Min Chiu, Hsiu-Yung Pan and Fu-Jen Cheng

Table S1. Demographic characteristics and diagnoses during study period.

All	Pre-COVID	During-COVID
Demographic characteristics of patients	*n* = 8991	*n* = 1405
Age (mean ± SD)	4.5 ± 3.7	4.7 ± 3.8
Male	4947	763
Past medical history of respiratory disease	221	31
Pneumonia	816	139
Upper respiratory tract infection	5230	863
Bronchitis	2311	325
Asthma	634	78

(A)

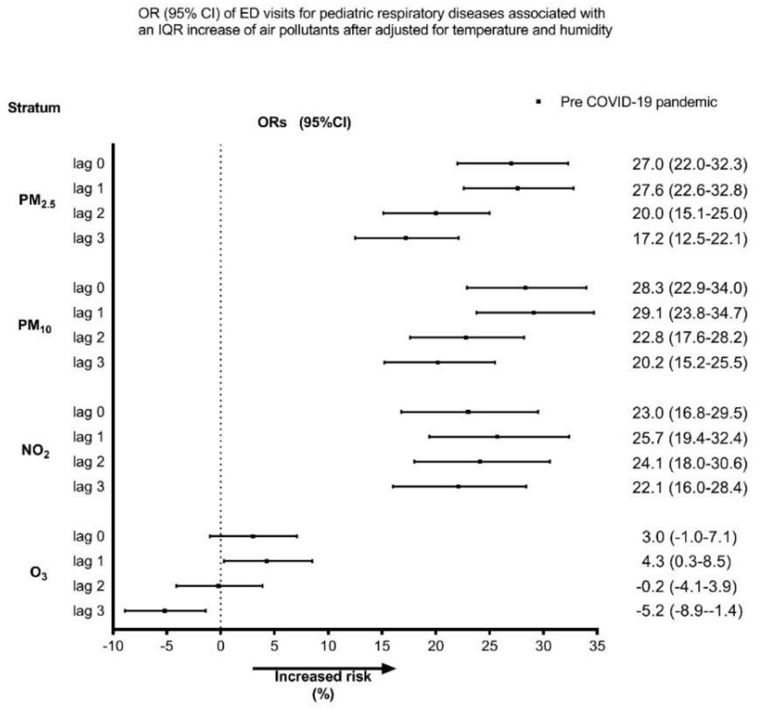

(B)

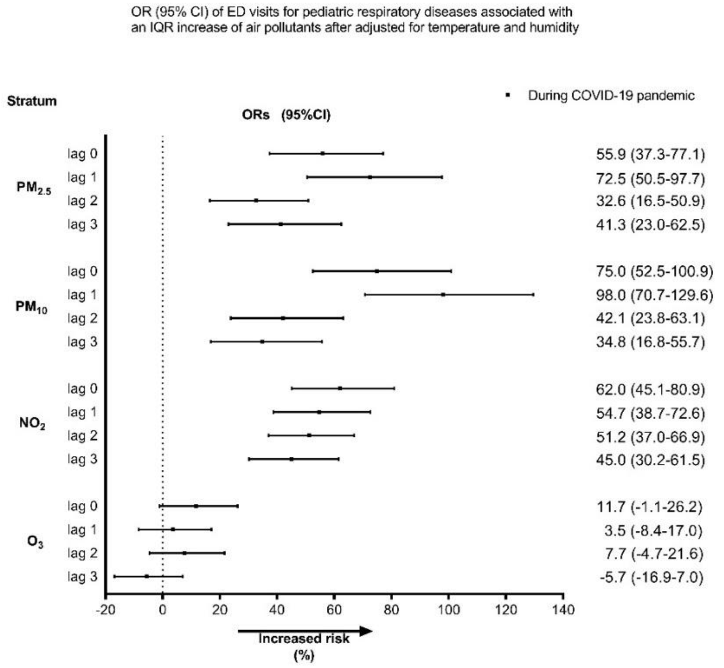

Figure S1. confidence intervals (CIs) for pediatric respiratory dis-ease-related ED visits associated with IQR increments in each air pollutant, pre- COVID-19 pandemic (**A**) and during the COVID-19 pandemic (**B**), with adjustments for temperature and humidity. ED, emergency depart-ment; IQR, interquartile range.
